# Trends and Inequities in Food, Energy, Protein, Fat, and Carbohydrate Intakes in Rural Bangladesh

**DOI:** 10.1093/jn/nxac198

**Published:** 2022-08-30

**Authors:** Akhter U Ahmed, M Mehrab Bakhtiar, Masum Ali, Julie Ghostlaw, Phuong Hong Nguyen

**Affiliations:** Poverty, Health, and Nutrition Division, International Food Policy Research Institute, Washington, DC, USA; Poverty, Health, and Nutrition Division, International Food Policy Research Institute, Washington, DC, USA; Poverty, Health, and Nutrition Division, International Food Policy Research Institute, Dhaka, Bangladesh; Poverty, Health, and Nutrition Division, International Food Policy Research Institute, Washington, DC, USA; Poverty, Health, and Nutrition Division, International Food Policy Research Institute, Washington, DC, USA

**Keywords:** Bangladesh, dietary diversity, dietary pattern, nutrient intake, South Asia

## Abstract

**Background:**

Tracking dietary changes can inform strategies to improve nutrition, yet there is limited evidence on food consumption patterns and how disparities in food and nutrient intakes have changed in Bangladesh.

**Objectives:**

We assessed trends and adequacies in energy and macronutrient intakes and evaluated changes in inequities by age group, sex, and expenditure quintile.

**Methods:**

We used panel data from the 2011 and 2018 Bangladesh Integrated Household Survey (*n* = 20,339 and 19,818 household members aged ≥2 y, respectively). Dietary intakes were collected using 24-h recall and food-weighing methods. Changes in energy and macronutrient intakes were assessed using generalized linear models and adjusted Wald tests. Inequities in outcomes were examined by age group, sex, and expenditure quintile using the Slope Index of Inequality and Concentration Index.

**Results:**

Between 2011 and 2018, dietary diversity improved across sex and age groups (30–46% in children, 60–65% in adolescents, 37–87% in adults), but diets remain imbalanced with ∼70% of energy coming from carbohydrates. There were declines in intakes of energy (3–8%), protein (3–9%), and carbohydrate (9–16%) for all age groups (except children aged 2–5 y), but an increase in fat intake (57–68% in children and 22–40% in adults). Insufficient intake remained high for protein (>50% among adults) and fat (>80%), whereas excessive carbohydrate intake was >70%. Insufficient energy, protein, and fat intakes, and excessive carbohydrate intakes, were more prevalent among poor households across survey years. Inequity gaps decreased for insufficient energy intake in most age groups, remained stable for insufficient protein intake, and increased for insufficient fat and excessive carbohydrate intakes.

**Conclusions:**

Despite improvements in dietary diversity, diets remain imbalanced and inequities in insufficient energy, protein, and fat intakes persist. Our findings call for coherent sets of policies and investments toward a well-functioning food system and social protection to promote healthier, more equitable diets in rural Bangladesh.

## Introduction

Adequate food and nutrient intakes are important determinants of health, well-being, and survival. The UNICEF conceptual framework highlights the influential role of age-appropriate, nutrient-rich food on maternal and child nutrition (1). Poor nutrient intakes predispose children to numerous adverse outcomes such as increased risks of anemia from iron deficiency, micronutrient deficiencies, growth retardation, inflammation, infectious disease, impaired memory, and overweight and obesity (2, 3). Furthermore, suboptimal diets have reverberating impacts on nutrition and health across the life span, representing the leading risk factor globally for chronic disease, such as heart disease, stroke, and type 2 diabetes (4, 5).

An aggregate view of dietary intakes of key foods and nutrients indicates that poor-quality diets are universal. In 2019, data from the Global Dietary Database show that, on average, adults 20 y and over in most countries did not consume the recommended intake of key foods, irrespective of income level (6). There are shortfalls in intakes observed for fruits, vegetables, legumes, nuts, and whole grains, whereas red meat is excessively consumed (6). Despite substantial improvements in food availability in Bangladesh in recent decades, diets remain monotonous (7), largely because the high cost of healthy diets is prohibitive to resource-constrained households (8). The lack of dietary diversity has contributed to the persistence of multiple micronutrient deficiencies across the life course, particularly among women and children in Bangladesh. Anemia is pervasive, affecting 37% of women of reproductive age and 44% of children under 5 y (9, 10). Other micronutrient deficiencies are also prevalent, including vitamin B-12 (22% in women), zinc (57% in women, 44% in children), and vitamin A (21% in children) (11).

Detailed food consumption data are important for monitoring progress on Sustainable Development Goals and other global targets (12). However, substantial data gaps remain, largely because collecting individual-level dietary intake is time-consuming, expensive, and presents several logistical and practical challenges (13, 14). Consequently, little is known about dietary intakes and inequities across the life course in Bangladesh and how these trends have changed over time. The Bangladesh Demographic and Health Surveys (BDHS) are conducted every 3–5 y since the early 1990s and contain nationally representative data on population, health, and nutrition, including food groups that can be used to calculate child feeding indicators (for children <5 y) (15). However, the BDHS does not collect individual-level food intake data. On diet-related topics, the Household Income and Expenditure Surveys (HIES) of Bangladesh collect data on the quantity, source, and value of food consumption at the household level. Although household-level food consumption data are relatively accurate at estimating individual-level nutrient intake and adequacy (16), the predictions are less accurate for certain populations, such as young children (17). It also cannot be used to estimate varying nutritional demands by age and physical status. Studies that examined individual nutrient intakes in Bangladesh were either constrained to subsets of the population or particular geographic areas, limiting the external validity. Other key populations such as adolescents are also often overlooked by these studies unless they are pregnant.

The Bangladesh Integrated Household Survey (BIHS) is the most comprehensive, nationally representative panel data of households in rural Bangladesh. It collected comprehensive information covering many aspects of livelihoods and health, including individual-level dietary intake for all household members. Previous studies used 1 cross-section of the BIHS (1) to measure energy and nutrient intakes among women of reproductive age or to explore how intra-household calorie inequities are linked to household characteristics in rural Bangladesh (18, 19). Since these studies used 1 round of cross-sectional data, they do not assess changes in food and nutrient intakes over time.

In this study, we examine the trends and adequacies in energy, protein, fat, and carbohydrate intakes between 2011 and 2018 in rural Bangladesh for all household members and assess changes in inequities in nutrient intakes by age group, sex, and expenditure quintile over the 7-y period.

## Methods

### Data source and study population

We used 2 rounds of BIHS data collected in 2011 and 2018 (20, 21). Detailed survey sampling procedures and questionnaires are available elsewhere (22). Briefly, the sampling used a 2-stage design. The first stage involved selecting 275 primary sampling units (PSUs; or villages) using the sampling frame developed from the 2001 population census of Bangladesh. Within each of the 7 strata representative of the 7 administrative divisions of the country, villages were selected from the sampling frame with probability proportional to population size. The second stage involved a village census for household listing and 20 households were randomly selected from each PSU from the list of all households in the PSU. This yielded 5503 households in total and 20,339 individuals in different age groups. The 2018 BIHS surveyed the same households from the 2011 survey using tracking information, such as address, GPS coordinates, and description of surrounding areas. Due to attrition, household merges, and household splits, the total numbers of households and individuals under the 2018 endline survey were 5604 and 19,818, respectively.

The Bangladesh Ministry of Food authorized the 2011 BIHS and the Bangladesh Ministry of Agriculture approved the BIHS 2018. Both survey rounds received ethical approval from the International Food Policy Research Institute, USA. Oral consent was obtained from the primary male and female respondents.

### Assessment of food and nutrient intakes

Data on food consumption for all household members were collected using a combination of 24-h food recall and food-weighing methods. Enumerators asked the household member with primary responsibility for preparing and distributing meals in the household—usually the primary adult female—about what food items were prepared on the previous day, the recipes prepared, the ingredients for the recipes, the sources of these ingredients, and the amounts of food eaten by various family members and guests. The enumerator asked the respondent to show the raw food ingredients and the amount of each ingredient, then weighed them using an electronic dietary scale with a precision of 1 g. The enumerator then asked the respondents to show the amount of food after cooking and weighed the amount by measuring cups and standard bowls as appropriate. The enumerator then asked the respondent about how much of the cooked food was distributed to each of the household members and each guest, if any. Several visual aids (e.g., standard pots, plates, bowls, cups, spoons, etc.) were used to help the respondent estimate the portion sizes distributed. In addition, individual-level information was collected on leftovers/recipes eaten from the previous day, meals taken away from home, food given away, and food fed to animals. Because of the gender-sensitive nature of interactions with outsiders in Bangladesh, interviews were conducted by male and female enumerators for male and female respondents of each selected household, respectively. All enumerators were extensively trained by participating in hands-on, practical sessions. A pretest and a survey pilot test were also conducted.

For adults and adolescents, all reported foods were categorized into 10 food groups based on the Minimum Dietary Diversity for Women Measurement guidelines (23), and minimum dietary diversity was defined as consuming 5 or more food groups within the last 24 h. For children aged <10 y, foods were categorized into 7 food groups based on WHO guidelines (24), and minimum dietary diversity was defined as consuming at least 4 food groups/d. No dietary diversity indicator has been validated for children aged 2–10 y.

The food ingredients were matched with the food list of the food composition table for Bangladesh (25) to obtain each item's energy, protein, fat, and carbohydrate content. Nutrition information for a few ingredients that were missing in the Bangladesh food composition table was taken from the USDA National Nutrient Database (26).

### Assessment of insufficient energy and macronutrient intakes

Estimated energy requirements (EERs) for adults were calculated for each individual following the methodology given in FAO/WHO's Human Energy Requirements Report (27). The EER of an individual was calculated by taking the product of the individual's basal metabolic rate (BMR) and his/her physical activity level (PAL). BMR was estimated from an individual's body weight using a set of standard predictive equations based on sex and age (**Supplemental Table 1**). PAL—the total energy required over 24 h divided by the BMR over 24 h—was categorized into 3 levels, as defined in FAO/WHO (2004): 1.4 for light, 1.7 for moderate, and 2.0 for high (27). The BIHS collected data on the main occupation for each household member. From these data, individuals were assigned a PAL value based on their main occupation reported in each survey round. Energy intake was categorized as insufficient if it was <85% of EER.

The percentage of energy from protein, carbohydrate, and fat intakes was compared with the Acceptable Macronutrient Distribution Ranges (AMDRs) recommended by the US Institute of Medicine for assessing insufficient or excessive intake (28). The AMDR is the percentage of energy intake that is associated with reduced risk of chronic disease yet provides adequate amounts of essential nutrients. A diet is considered balanced if the contributions of protein, fat, and carbohydrate to energy for an individual meet the AMDR for a specific age group and sex. Consuming below or above these ranges implies increased risk of insufficient intakes of essential nutrients or chronic disease. For adults, the AMDRs for fat, carbohydrate, and protein are 20–35%, 45–65%, and 10–35%, respectively. AMDRs for children aged 1–3 y are 30–40%, 45–65%, and 5–20%, respectively; and AMDRs for children aged 4–18 years are 25–35%, 45–65%, and 10–30%, respectively.

### Other variables

The age of the respondent was collected by asking the date of birth for children and complete years for the adolescent and adults. Age was categorized into 6 groups based on physical growth and nutrient requirements: *1*) 2 to <5 y (preschool education), *2*) 5 to <10 y (school age), *3*) 10–18 y (adolescents), *4*) 19–40 y (early and middle adulthood), *5*) 41–60 y (late adulthood), and *6*) ≥61 y (old age).

Economic inequity among sample households was assessed from the distribution of household income. Our analysis used the household consumption expenditure as a proxy for income for 2 reasons. First, expenditures are likely to reflect permanent income and, hence, are a better indicator of consumption behavior (29). Second, consumption expenditure data are more widely used for measuring poverty than income data because of the difficulty in accurately measuring income. Expenditure data have been reported to be less prone to error, easier to recall, and more stable over time than income data (30). Since expenditures are intended to serve as a proxy for income, the term “income” is used to represent consumption expenditures.

The measure of total consumption expenditure is quite extensive and draws upon responses to several sections of the household survey. In brief, consumption is measured as the sum of total food consumption and total nonfood (nondurable and durable) expenses. Expenditures on individual consumption items were aggregated to construct total monthly expenditures. Quantities of goods produced by the household for home consumption were valued at the average unit market prices of commodities. Households were then categorized into quintiles based on per capita expenditure, where the lowest quintile (Q1) represents the poorest 20% and the highest quintile (Q5) represents the richest 20% of the pooled population.

### Statistical analysis

Estimates of energy and nutrient intakes were tested for normality using Shapiro–Wilk tests. Because most distributions of nutrient intakes were skewed, both mean (SD) and median intakes were reported. All nutrients were log-transformed for statistical testing.

Descriptive statistics reported food, energy, and nutrient intakes for different age groups by sex. Graphical methods were used to visualize insufficient intakes in both children and adults and by sex over time. The statistical significance of changes between 2011 and 2018 was tested using generalized linear models for continuous variables and adjusted Wald tests for categorical variables.

To examine inequities in energy and nutrient intakes by expenditure quintile within each age group, equity plots were used to visualize the prevalence of insufficient intake in 2011 and 2018, disaggregated by per capita expenditure quintile for each age/sex group. Then, absolute and relative income inequities for each outcome were examined by age group using the slope index of inequality (SII) and the concentration index (CIX) (28, 29). The SII represents the absolute differences by percentage points (pp) in the estimated values of the nutrient intake between the lowest and highest expenditure quintiles, and CIX measures the inequality that is related to the Gini coefficient. The range of values for both SII and CIX is –100 to +100, where positive values indicate that the outcome is more prevalent among the highest expenditure quintile and negative values suggest that the outcome is more common among the poorest expenditure quintile. These 2 complex measures are widely used to summarize health inequity in a series of subgroups such as expenditure quintiles, accounting for the entire distribution of outcomes over the 5 quintiles and weighted by the sample size of each quintile. These 2 inequity measures were also used to assess changes in inequities over time.

All models were adjusted for cluster sampling design and survey sampling weights. Two-sided *P* < 0.05 was used for statistical significance. All analyses were performed using Stata version 16 (StataCorp).

## Results

### Household characteristics

The average household size slightly declined from 4.1 persons in 2011 to 4.0 persons in 2018 (**Table 1**). The highest concentration of people was in the 19–40-y-age group in both 2011 (34%) and 2018 (30%). Approximately 40% of the sample population was under age 18 and approximately 9% of people were over age 60 in 2018. The dependency ratio estimates suggest that, in 2018, 72% of working-aged people had non–working-age household members (0–14 y and ≥61 y) to support.

**TABLE 1:** Household characteristics

	2011		2018		Change from 2011 to 2018, %
	*n*	%	*n*	%	
Household size (mean person)	5503	4.1	5604	4.0	−2.4
Demographic composition by age group					
0–1 y	999	4.3	925	4.0	−7.4
2 to <5 y	1513	6.5	1435	6.2	−5.2
5 to <10 y	2969	12.8	2660	11.5	−10.4
10–18 y	4517	19.5	4497	19.4	−0.4
19–40 y	7768	33.6	6925	29.9	−10.9
41–60 y	3929	17.0	4551	19.7	15.8
≥61 y	1440	6.2	2144	9.3	49.3
Dependency ratio^1^	5503	73.4	5604	72.3	−1.5
Education					
Net primary school enrollment of boys^2^	5503	80.0	5604	89.8	12.3
Net primary school enrollment of girls^2^	5503	82.9	5604	92.5	11.6
Net secondary school enrollment of boys^3^	5503	36.8	5604	50.0	35.9
Net secondary school enrollment of girls^3^	5503	46.1	5604	62.1	34.7
Years of schooling, male household head	5503	3.3	5604	3.7	12.1
Years of schooling, wife of male household head	5503	3.1	5604	3.8	22.6
Years of schooling of adult male ≥18 y	5503	4.0	5604	4.6	15.0
Years of schooling of adult female ≥18 y	5503	3.5	5604	4.3	22.9
No schooling adult male	5503	42.8	5604	36.1	-15.7
No schooling adult female	5503	46.6	5604	37.8	-18.9
Economic status					
Per capita expenditure in constant 2005/2006 prices (*taka*/mo)	5503	1554	5020	1803	16.0
Share of food expenditure in total expenditure	5503	57.7	5020	52.7	−8.7
Prevalence of extreme poverty^4^	5503	17.4	5020	9.2	−47.0
Average owned cultivable landholding size	5503	45.8	5604	42.7	−6.8
Landlessness^5^	5503	54.8	5604	54.9	0.2

1 Dependency ratio = number of dependents (<15 or >60 y of age) divided by number of working age people (15–60 y).

2 Net primary school enrollment rate = all primary school–going children aged 6–10 y/all children aged 6–10 y.

3 Net secondary school enrollment rate = all secondary school–going children 11–17 y/all children aged 11–17 y.

4 Percentage of people living on <$1.90/d international poverty line adjusted to 2011 purchasing power parity (PPP) exchange rate. These estimates are obtained from reference 43, which used the same Bangladesh Integrated Household Survey (BIHS) 2011 and 2018 datasets.

5 Percentage of people who do not own any cultivable land except for homestead land. The size of landholding is measured in decimals (100 decimals = 1 acre).

The rate of school enrollment among primary and secondary school-age boys and girls increased from 2011 to 2018. Educational attainment in terms of years of schooling of adult family members also increased. Despite these improvements, more than one-third of adults (36% of adult males and 38% of adult females) never attended school as of 2018. Average per capita daily expenditures (as a proxy for income) in constant 2005/2006 prices increased by 16% from 2011 to 2018 and extreme poverty declined by 47% in the same period. Although land is the most important factor of agricultural production, more than half (∼55%) of households did not own any cultivable land in both 2011 and 2018.

### Changes in food intakes

Between 2011 and 2018, minimum dietary diversity significantly improved in both males and females for all age groups (30–46% in children, 60–65% in adolescents, and 37–87% in adults) (**Table 2**). This improvement was higher for females than males among groups aged 19–40 y (87 vs. 61%) and ≥61 y (75 vs. 37%). The changes in dietary diversity were mainly due to an increase in the proportion of individuals who consumed different food groups such as eggs, pulses/legumes, and dairy products (in both children and adults), as well as vegetables and nuts/seeds (in adults).

**TABLE 2:** Food-group consumption by age group, sex, and survey rounds^1^

	Male, %			Female, %		
	2011 (*n* = 9932)	2018 (*n* = 9536)	Change from 2011 to 2018	2011 (*n* = 11,054)	2018 (*n* = 10,787)	Change from 2011 to 2018
2 to <5 y						
Grains, roots, or tubers	100.0	100.0	0.0	100.0	100.0	0.0
Vitamin A–rich plant foods	33.5	38.0	13.4	32.2	38.3	18.9*
Other fruits or vegetables	98.5	99.0	0.5	98.1	99.4	1.3
Meat, poultry, fish, seafood	72.3	72.8	0.7	73.0	73.6	0.8
Eggs	10.9	24.3	122.9***	13.2	26.2	98.5***
Pulses/legumes/nuts	20.7	32.5	57.0***	23.3	32.1	37.8*
Milk and milk products	20.1	35.4	76.1***	19.7	34.7	76.1***
Minimum dietary diversity (≥4 food groups)	47.5	69.5	46.3***	51.2	69.8	36.3***
5 to <10 y						
Grains, roots, or tubers	100.0	100.0	0.0	100.0	100.0	0.0
Vitamin A–rich plant foods	39.9	39.4	−1.3	40.5	40.0	−1.2
Other fruits or vegetables	99.5	99.9	0.4	99.7	99.9	0.2
Meat, poultry, fish, seafood	75.8	76.9	1.5	73.5	76.1	3.5
Eggs	10.2	24.3	138.2***	11.4	22.9	100.9***
Pulses/legumes/nuts	19.6	33.3	69.9***	19.8	34.1	72.2***
Milk and milk products	19.1	29.2	52.9***	17.8	23.8	33.7*
Minimum dietary diversity (≥4 food groups)	52.3	67.7	29.4***	51.7	66.6	28.8***
10–18 y						
Grains, white roots and tubers, and plantains	100.0	100.0	0.0	100.0	100.0	0.0
Pulses (beans, peas, and lentils)	21.9	33.7	53.9***	21.8	34.7	59.2***
Nuts and seeds	1.3	4.4	238.5***	1.6	4.2	162.5*
Dairy	17.9	23.8	33.0**	18.8	22.8	21.3*
Meat, poultry, and fish	77.9	79.3	1.8	77.4	77.4	0.0
Eggs	11.4	22.1	93.9***	11.3	21.1	86.7***
Dark-green leafy vegetables	40.2	42.9	6.7	43.4	44.9	3.5
Other vitamin A–rich fruits and vegetables	1.1	4.0	263.6***	1.7	2.4	41.2^c^
Other vegetables	99.6	100	0.4	99.9	99.9	0.0
Other fruits	10.2	18.3	79.4***	11.0	24.2	120.0^***,b^
Minimum dietary diversity (≥5 food groups)	23.7	39.0	64.6***	25.1	40.2	60.2***
19–40 y						
Grains, white roots and tubers, and plantains	100.0	100.0	0.0	100.0	100.0	0.0
Pulses (beans, peas, and lentils)	22.4	37.9	69.2***	20.5	32.7	59.5^***,a^
Nuts and seeds	1.1	4.1	272.7***	1.2	4.3	258.3***
Dairy	19.4	24.2	24.7***	14.9	23.7	59.1^***,b^
Meat, poultry, and fish	79.1	80.3	1.5	75.8	79.7	5.1^*,a^
Eggs	12.4	23.5	89.5***	10.9	21.6	98.2***
Dark-green leafy vegetables	38.6	40.9	6.0	39.4	43.8	11.2*
Other vitamin A–rich fruits and vegetables	1.1	2.8	154.5***	1.2	2.9	141.7***
Other vegetables	99.5	99.8	0.3	99.8	100.0	0.2
Other fruits	7.4	10.8	45.9***	8.8	16.9	92.0^***,c^
Minimum dietary diversity (≥5 food groups)	23.8	38.2	60.5***	20.4	38.2	87.3^***,a^
41–60 y						
Grains, white roots and tubers, and plantains, *%*	100.0	100.0	0.0	100.0	100.0	0.0
Pulses (beans, peas, and lentils)	23.8	37.0	55.5***	22.5	35.7	58.7***
Nuts and seeds	1.1	3.0	172.7**	1.1	3.7	236.4***
Dairy	22.8	24.5	7.5	17.9	23.5	31.3^*,a^
Meat, poultry, and fish	78.2	77.8	−0.5	78.2	77.8	−0.5
Eggs	12.2	21.3	74.6***	10.0	16.8	68.0***
Dark-green leafy vegetables	39.0	41.9	7.4	43.9	44.6	1.6
Other vitamin A–rich fruits and vegetables	1.3	2.7	107.7*	1.5	2.9	93.3*
Other vegetables	99.8	99.9	0.1	99.4	99.9	0.5
Other fruits	8.0	12.1	51.3**	9.4	15.8	68.1***
Minimum dietary diversity (≥5 food groups)	25.2	36.0	42.9***	24.5	37.4	52.7***
≥61 y						
Grains, white roots and tubers, and plantains	100.0	100.0	0.0	100.0	100.0	0.0
Pulses (beans, peas, and lentils)	22.3	36.7	64.6***	20.8	35.4	70.2***
Nuts and seeds	1.9	4.1	115.8*	1.3	3.9	200.0*
Dairy	24.0	24.9	3.7	20.2	25.1	24.3
Meat, poultry, and fish	79.7	78.1	−2.0	77.0	76.6	−0.5
Eggs	10.6	19.4	83.0***	9.2	19.4	110.9***
Dark-green leafy vegetables	41.5	42.3	1.9	40.1	44.3	10.5
Other vitamin A– rich fruits and vegetables	1.8	3.2	77.8	1.5	2.9	93.3
Other vegetables	99.7	99.8	0.1	99.4	99.7	0.3
Other fruits	10.3	11.3	9.7	7.3	11.1	52.1*
Minimum dietary diversity (≥5 food groups)	26.5	36.4	37.4**	21.4	37.4	74.8^***,a^

1 ^*,**,***^Significant difference for changes between 2011 and 2018: *P < 0.5, **P < 0.01, ***P < 0.001. Letters indicate significant difference for changes from 2011 to 2018 between males and females: ^a^*P* < 0.5, ^b^*P* < 0.01, ^c^*P* < 0.001.

Average quantities of total and some specific commonly consumed food at the household level in 2011 and 2018 are presented in **Supplemental Table 2**. Between 2011 and 2018, the total quantity of food consumed decreased by 6%, from 930 to 873 g/d. These quantities are much lower than the desirable dietary intake for the Bangladeshi population of 1240 g/d (31). Rice is the main staple food in Bangladesh, which is consumed in the largest amounts, followed by other vegetables and potatoes. Average rice consumption per capita per day decreased by 15% over the 7-y period, indicating that diets are becoming less rice-centric and more diverse. Between 2011 and 2018, per capita daily consumption of eggs doubled, fruits increased by 143%, milk and milk products by 61%, and meat by 38%. Conversely, consumption of potatoes, green leafy vegetables, fish, and sweeteners (sugar) declined. Compared with the desirable intake for the Bangladeshi population (31), the quantity intakes (grams/day) in 2018 were lower for pulses (14 vs. 50), eggs (10 vs. 30), meat (20 vs. 40), fish (47 vs. 60), milk and milk products (28 vs. 130), fruits (20 vs. 100), and green leafy vegetables (31 vs. 100). However, despite a decline in rice consumption between 2011 and 2018, the average per capita daily consumption remained higher than the desirable intake in 2018 (379 vs. 350).

### Changes in energy intakes

**FIGURE 1:**
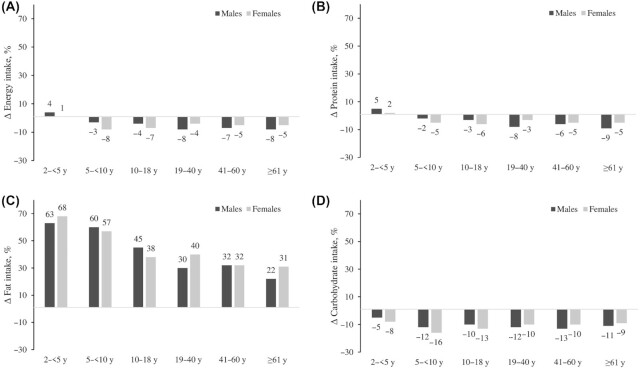
Changes in energy and macronutrient intakes between 2011 and 2018, by age group and sex. Changes in energy intakes (A), protein intakes (B), fat intakes (C), and carbohydrate intakes (D) are shown.

Energy intakes did not change between 2011 and 2018 among children aged <5 y, but decreased by 3–8% among children aged >5 y and adults over the 7-y period (**Table 3** and **Figure 1**A). Among adults aged >19 y, the reduction was slightly higher for men (7–8%) than for women (4–5%). The proportion of insufficient energy intake (<85% EER) was high in children aged <5 y (∼40%) and did not change over time (**Table 4**). In contrast, the proportion of insufficient energy intake was lower in other age groups and increased over time. For example, insufficient energy intake was 14% in males aged 5–18 y in 2011 and increased by 8–11 pp in 2018. Similar increases were observed among men aged >19 y (by 10–12 pp). Insufficient energy intakes remained stable at 27–28% among women aged 19–60 y, but doubled among girls aged 10–18 y (from 20% to 42%), and increased by 10 pp (from 13% to 23%) among women aged ≥61 y.

**TABLE 3:** Energy and macronutrient intakes by age group, sex, and survey rounds^1^

	Male					Female				
	2011/12 (*n* = 9584)		2018/19 (*n* = 9267)		Ratio (95% CI)	2011 (*n* = 10,755)		2018 (*n* = 10,549)		Ratio (95% CI)
	Mean (SD)	Median	Mean (SD)	Median		Mean (SD)	Median	Mean (SD)	Median	
Energy, kcal/d										
2 to <5 y	1014 (403)	961	1028 (373)	978	1.04 (0.98–1.07)	977 (374)	962	965 (335)	917	1.01 (0.96–1.07)
5 to <10 y	1514 (454)	1468	1461 (441)	1405	0.97 (0.94–0.99)	1494 (436)	1470	1383 (424)	1311	0.92^***,a^ (0.90–0.95)
10–18 y	2216 (618)	2139	2134 (645)	2061	0.96 (0.93–0.98)	2048 (538)	1988	1897 (514)	1843	0.93^***,a^ (0.90–0.94)
19–40 y	2780 (637)	2706	2561 (656)	2508	0.92 (0.90–0.93)	2364 (555)	2312	2261 (533)	2219	0.96^***,c^ (0.94–0.97)
41–60 y	2685 (649)	2600	2508 (616)	2433	0.93 (0.91–0.95)	2254 (557)	2193	2137 (523)	2090	0.95*** (0.93–0.97)
≥61 y	2352 (641)	2323	2185 (639)	2155	0.92 (0.89–0.95)	1943 (518)	1893	1856 (531)	1828	0.95** (0.92–0.99)
Protein, g/d										
2 to <5 y	25.8 (12.3)	24.4	26.1 (10.2)	24.4	1.05 (0.99–1.11)	24.7 (11.2)	22.5	24.5 (9.7)	22.2	1.02 (0.97–1.08)
5 to <11 y	37.8 (15.0)	34.9	36.8 (13.2)	34.6	0.98 (0.95–1.01)	36.8 (13.6)	35.3	34.8 (12.8)	32.9	0.95** (0.91–0.98)
11 to <20 y	54.6 (18.6)	51.7	53.4 (18.9)	50.3	0.97 (0.95–1.00)	51.0 (18.1)	48.3	47.5 (15.6)	44.9	0.94^***,a^ (0.91–0.96)
20 to <41 y	69.1 (21.4)	65.5	63.6 (19.4)	60.8	0.92 (0.90–0.94)	58.3 (19.2)	55.1	56.2 (16.8)	53.7	0.97^***,c^ (0.95–0.99)
41 to <61 y	66.6 (21.1)	63.1	62.5 (19.3)	59.9	0.94 (0.92–0.96)	56.4 (18.4)	53.8	53.4 (16.6)	50.9	0.95*** (0.93–0.97)
≥61 years	60.3 (21.7)	56.7	54.9 (19.3)	52.7	0.91 (0.88–0.95)	49.3 (17.1)	47.0	46.9 (16.0)	45.4	0.95** (0.91–0.99)
Fat, g/d										
2 to <5 y	15.3 (12.7)	12.2	22.7 (12.6)	20.1	1.63 (1.50–1.78)	13.8 (10.3)	11.2	21.1 (12.4)	19.0	1.68 (1.53–1.85)
5 to <11 y	19.2 (13.4)	15.9	28.6 (15.6)	25.8	1.60 (1.51–1.69)	18.0 (11.8)	15.5	27.0 (15.5)	24.2	1.57*** (1.48–1.67)
11 to<20 y	25.8 (17.0)	22.0	35.9 (20.7)	31.5	1.45 (1.38–1.52)	24.3 (16.3)	20.3	31.6 (16.9)	28.7	1.38*** (1.31–1.45)
20 to<41 y	33.8 (21.2)	28.7	42.2 (22.2)	37.8	1.30 (1.24–1.35)	27.7 (17.9)	23.2	36.8 (19.2)	33.1	1.40^***,c^ (1.35–1.45)
41 to <61 y	32.2 (19.9)	27.3	40.5 (21.1)	36.3	1.32 (1.26–1.38)	27.3 (17.3)	23.2	34.5 (18.1)	31.0	1.32*** (1.27–1.37)
≥61 y	29.9 (19.5)	25.9	35.1 (18.6)	31.7	1.22 (1.14–1.30)	23.9 (14.9)	19.9	30.6 (18.4)	26.1	1.31*** (1.21–1.41)
Carbohydrate, g/d										
2 to <5 y	188 (75.7)	177	175 (68.4)	167	0.95 (0.90–1.01)	183 (71.7)	179	165 (59.9)	157	0.92 (0.87–0.98)
5 to <11 y	288 (86.4)	278	256 (82.1)	246	0.88 (0.86–0.91)	287 (84.6)	285	243 (77.6)	231	0.84^***,b^ (0.81–0.87)
11 to <20 y	427 (120)	413	387 (122)	371	0.90 (0.87–0.92)	393 (101)	385	344 (98.1)	332	0.87*** (0.85–0.89)
20 to <41 y	532 (122)	521	466 (126)	458	0.87 (0.85–0.88)	455 (105)	444	412 (104)	401	0.90^***,c^ (0.88–0.92)
41 to <61 y	515 (125)	499	458 (119)	445	0.88 (0.87–0.90)	431 (102.9)	421	390 (99.2)	381	0.90*** (0.88–0.92)
≥61 y	445 (120)	440	399 (121)	387	0.89 (0.86–0.92)	370 (100.9)	357	336 (100)	334	0.91*** (0.87–0.94)

1 ^**,***^Significant difference for changes between 2011 and 2018: ^**^P < 0.01, ***P < 0.001. Letters indicate significant difference for changes from 2011 to 2018 between males and females: ^a^*P* < 0.5, ^b^*P* < 0.01, ^c^*P* < 0.001.

**TABLE 4:** Proportion of insufficient intake for energy, protein, and fat and excessive intake of carbohydrate by age group, sex, and survey rounds^1^

	Male			Female		
	2011 (*n* = 9584)	2018 (*n* = 9267)	*P*	2011 (*n* = 10,755)	2018 (*n* = 10,549)	*P*
Energy, % insufficient						
2 to <5 y	42.3	39.6	0.4029	35.9	39.4	0.2999
5 to <10 y	14.3	22.1	0.0001	12.4	21.8	0.0001
10–18 y	14.4	25.4	0.0001	20.3	42.1	0.0001
19–40 y	19.6	28.9	0.0001	27.0	28.7	0.2401
41–60 y	21.9	34.0	0.0001	27.6	28.3	0.6958
≥61 y	10.7	21.0	0.0001	13.0	22.9	0.0001
Protein, % insufficient						
2 to <5 y	19.6	16.2	0.1400	19.7	18.0	0.473
5 to <10 y	60.8	54.0	0.0076	60.8	54.1	0.0066
10–18 y	62.0	56.4	0.0042	60.4	57.2	0.1048
19–40 y	58.7	58.2	0.771	63.4	57.9	0.0004
41–60 y	58.9	57.3	0.366	57.3	56.6	0.7372
≥61 y	53.6	55.2	0.582	55.1	52.6	0.4302
Fat, % insufficient						
2 to <5 y	92.7	86.1	0.0005	95.8	87.2	0.0001
5 to <10 y	96.7	84.4	0.0001	98.4	85.8	0.0001
10–18 y	98.3	92.2	0.0001	98.3	93.0	0.0001
19–40 y	94.2	82.4	0.0001	94.5	83.5	0.0001
41–60 y	94.3	82.6	0.0001	94.6	84.1	0.0001
≥61 y	92.4	83.0	0.0001	92.6	84.1	0.0001
Carbohydrate,^2^ % excessive						
2 to <5 y	85.4	68.6	0.0001	86.7	70.0	0.0001
5 to <10 y	94.0	76.3	0.0001	95.8	77.6	0.0001
10–18 y	95.9	84.7	0.0001	96.4	86.8	0.0001
19–40 y	95.4	87.5	0.0001	96	88.7	0.0001
41–60 y	96.1	88.0	0.0001	96.3	88.5	0.0001
≥61 y	93.7	88.3	0.0001	94.6	87.9	0.0001

1 Adequacies were classified as insufficient or excessive based on the Acceptable Macronutrient Distribution Ranges (AMDRs) from the Institute of Medicine's *Dietary Reference Intakes* guidance document (24). An individual is classified as having insufficient protein and fat intakes if consumption is less than the lower limit of that particular AMDR. These intake ranges vary by age. An individual is considered to have excessive carbohydrate intake if consumption is greater than the upper limit of the AMDR for carbohydrates. An individual is classified as having insufficient energy intake if energy makes up <85% of total estimated energy (kcal) requirement.

2 0–2% insufficient carbohydrate intake.

### Changes in protein intakes

Protein intakes remained stable among boys aged <18 y and girls aged 2–5 y but decreased by 6–9% among men and by 3–5% among women (Table 3 and Figure 1B). Approximately 20% of children aged <5 y and over half of older children and adults (52–62%) did not meet the protein intake requirements based on AMDRs. The proportion of insufficient protein intake remained high and did not change among adult males aged >18 y and women aged >41 y (Table 4). Insufficient intakes slightly decreased among boys aged 5–18 y (by 6–7 pp), girls aged 5–9 y (by 7 pp), and women aged 19–40 y (by 10 pp).

### Changes in fat intakes

Between 2011 and 2018, fat intakes substantially increased in both boys and girls, women and men, and increased more in children aged <10 y (57–68%) than in adults (22–40%) (Table 3 and Figure 1C). In 2011, most of the sample (>90%) did not meet the fat intake requirements. The proportion of insufficient fat intake decreased in all age groups for both males and females (5–13 pp) (Table 4).

### Changes in carbohydrate intakes

Similar to the trend in energy, carbohydrate intakes did not change between 2011 and 2018 among children <5 years old but decreased by 9–16% in other age groups (Table 3 and Figure 1D). Carbohydrate intakes exceeded the upper bound of the AMDRs for most of the population, and decreased for all age groups over time, with a higher reduction among children aged 2–9 y (17–18 pp) than adults (5–8 pp).

### Changes in balanced diet

**FIGURE 2:**
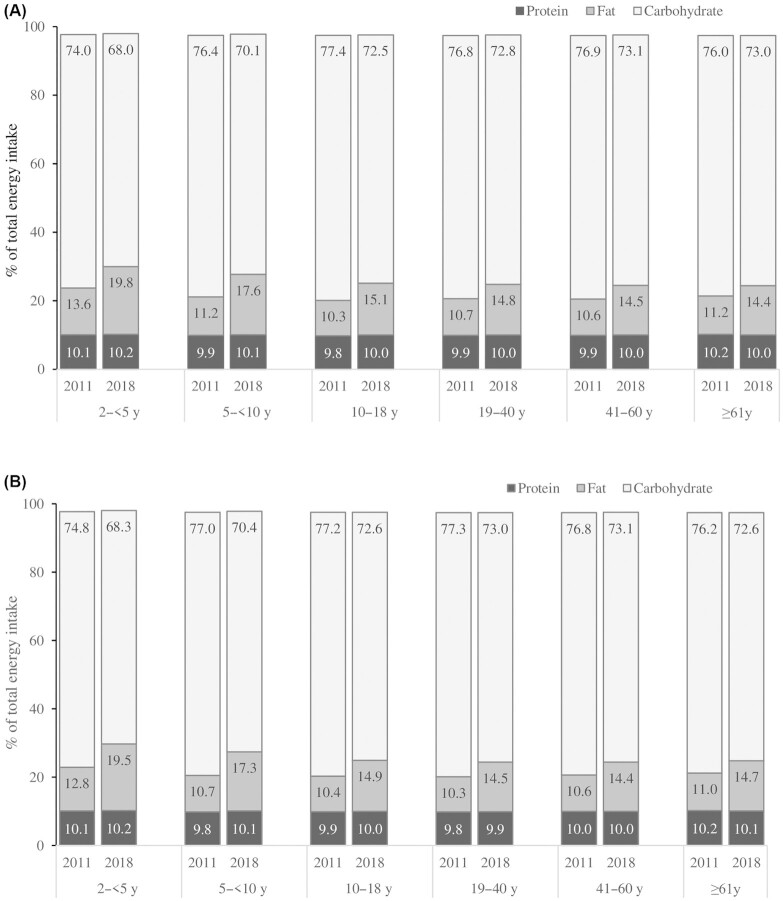
Contribution (%) of energy from protein, fat, and carbohydrate, by age group, sex, and survey rounds. Males (A), females (B).

In 2011, more than three-quarters of energy contributions came from carbohydrate, 10–14% from fat, and ∼10% from protein (**Figure 2**). The contribution of protein to total energy did not change over time, but the contribution of fat to total energy slightly increased (by 3–7% for different age groups) and the contribution of carbohydrate decreased accordingly.

The contribution of some of the most common foods to total protein, fat, and carbohydrate is shown in **Supplemental Table 3**. Rice is the main source of protein, contributing more than half of total protein, followed by fish, vegetables, and pulses. The contribution of rice to protein decreased over time (by 13%), whereas the contribution of pulses and animal-source foods to protein increased.

### Inequities in energy and macronutrient intakes

**FIGURE 3:**
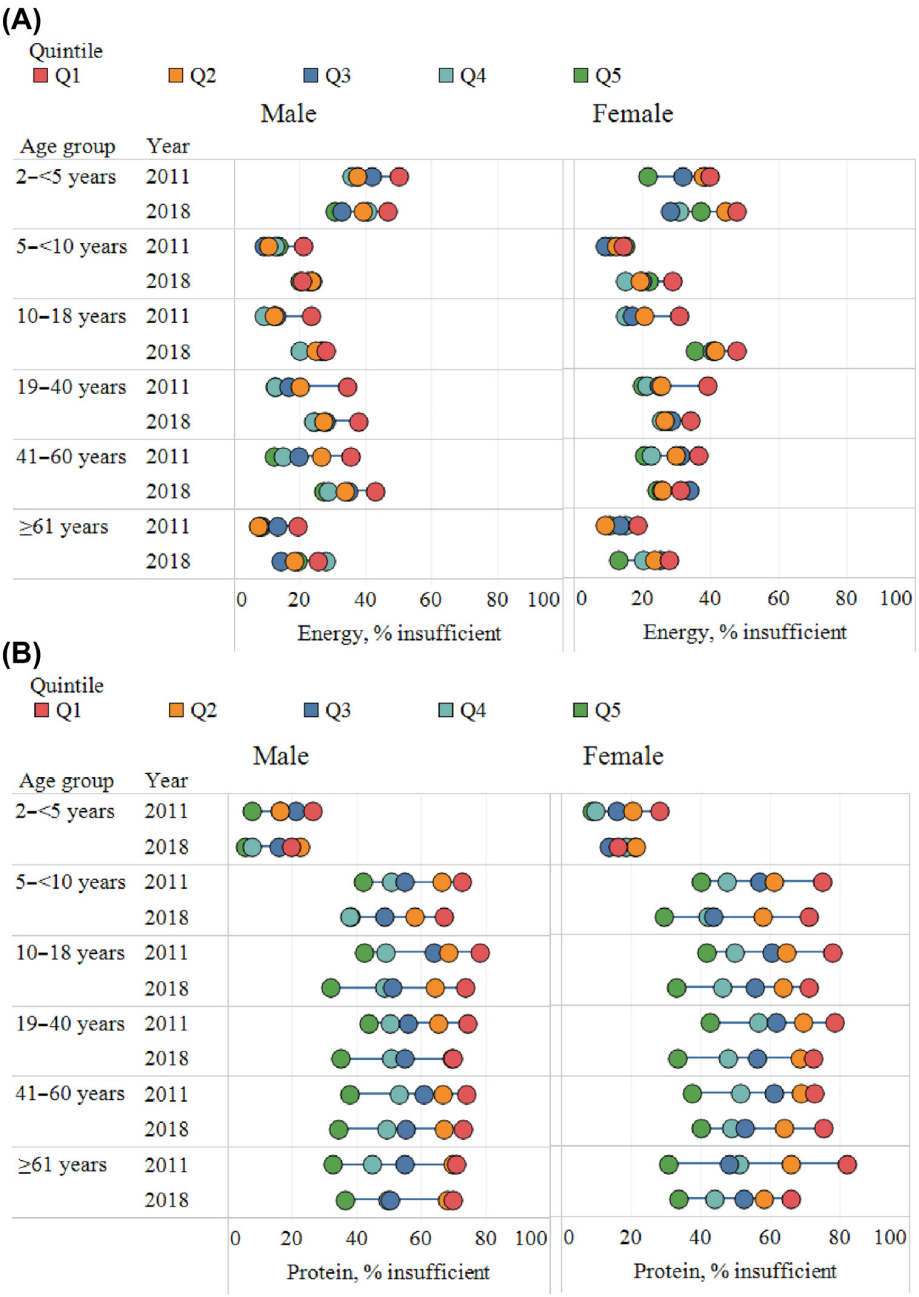
Inequity in energy and macronutrient intakes, by age group, sex, and survey rounds. Insufficient energy intake (A), protein intake (B), and fat intake (C) and excessive carbohydrate intake (D). Q, quintile.

Insufficient energy intake was higher among poorer compared with wealthier individuals across most age groups and survey years, as shown by the equity plots and negative SII and CIX (**Figure 3**A and **Supplemental Table 4**). Among males, the wealth gaps (Q5 vs. Q1) were found in all age groups in 2011 (SII ranged from –29 to –12 pp), except for men aged ≥61 y. The inequity gaps in energy intake decreased between 2011 and 2018 among boys 5–10 y old and men aged 19–60 y, but did not change (with statistical significance) for children <5 y old and men aged ≥61 y. The wealth gaps also decreased among women aged 19–60 y, but no statistical change was observed among girls aged <19 y or among the elderly aged ≥61 y.

Insufficient protein intakes were observed more among the poor than the rich across all age groups and survey years (Figure 3B and Supplemental Table 4). The wealth gaps in insufficient protein intakes were narrower among children <5 y old (17–27 pp) and wider among children 5–18 y old and adults (6–54 pp). Over time, the wealth gaps in protein intake did not change across age groups in both males and females, except among girls aged <5 y whose wealth gap decreased over time.

Insufficient fat intake was more prevalent among poorer compared with wealthier households, but the prevalence was high overall (>65%), even among individuals living in wealthier households (Figure 3C and Supplemental Table 4). The differences between income groups were medium in all age groups (in 2011—SII: −8 to −24 pp for males and −5 to −19 pp for females), but increased between 2011 and 2018 in both males (SII: −19 to −36 pp) and females (SII: −19 to −32 pp), except among children aged <5 y and the elderly aged ≥61 y.

In contrast with protein and fat intake, poorer individuals were more likely to have excessive carbohydrate intakes compared with the wealthier households (Figure 3D and Supplemental Table 4). The wealth gaps were large for children aged <5 y (−32 to −40 pp for girls and −42 to −47 pp for boys) and did not change over time. The wealth gaps in terms of excessive carbohydrate intakes grew over time for all age groups except for the elderly aged ≥61 y.

## Discussion

Food consumption in Bangladesh has been assessed since the 1970s using several rounds of HIES. However, these studies were based on household-level data; hence, these findings cannot be extrapolated to individual-level dietary intakes, particularly for women of reproductive age and children. Previous work has shown differences in intra-household patterns of food consumption and the role of gender bias in South Asia (32). To the best of our knowledge, this is the first study in a developing country in the last decade to investigate the trends and adequacies of food, energy, and macronutrient intakes at the level of household members. This study also focuses on the inequities in energy and macronutrient intakes by sex, expenditure quintile, and age groups.

Between 2011 and 2018, dietary diversity improved significantly for every age group, but diets remained imbalanced with approximately 70% of energy from carbohydrates. Energy, protein, and carbohydrate intakes decreased in all age groups (except for children 2–5 y old), whereas fat intake increased. Compared with wealthier households, individuals in poorer households had higher levels of insufficiency in energy, protein, and fat intakes, and excessively consumed carbohydrates. The wealth gaps decreased for insufficient energy intake in most age groups, were unchanged for insufficient protein intake, while they increased for insufficient fat and excessive carbohydrate intakes.

Improvements in dietary diversity observed in this study are consistent with recent evidence showing a greater proportion of individuals consumed fish, eggs, meat, milk, and vegetables (33, 34), and increasing dietary diversity among women of reproductive age (15–49 y) (35). Despite Bangladesh's rice-centric agriculture, production of other food including animal-sourced food (fish, meat, milk, and egg) have increased in the previous decade. However, when comparing to the desirable intake for Bangladeshi population (31), the quantity consumed of pulses, eggs, milk, and milk products accounted for only about one-third of the requirement, and meat intake is still only half of the requirement (Supplemental Table 2). These suboptimal intakes explain the high insufficient intake of protein (>50% among adults) and fat (>80%), and the imbalanced diet pattern despite improvements in dietary diversity. On the other hand, although rice consumption declined, the average consumption remained higher than the recommended level in 2018. Our findings are aligned with previous research showing that rice remains a major contributor of energy and macronutrients in the Bangladeshi diet (33).

Decreasing intakes of energy, protein, and carbohydrates and increasing fat intake observed under our study is similar to the official estimates of the Bangladesh HIES (36). Decreasing energy and protein intakes may be due to decreased consumption of rice and other cereals, which was not adequately compensated by higher consumption of vegetables, fish, meat, and other food groups. There was a 12–15% decrease in the consumption of rice and other cereals in our study population, similar to estimates from a previous study showing a decrease from 731.4 g/d per adult male equivalent (AME) to 683.4 g/d per AME from 1985 to 2016 in Bangladesh (37). Although a study using the FAO food balance sheet found an increasing trend in protein intake (38), per capita protein intake based on HIES decreased (by 6.4%) in rural areas from 2010 to 2016 (36), which is similar to our findings (6–9% among men and 3–5% among women). Again, rice is a major contributor of protein intakes in the rural Bangladesh diet (accounting for approximately half of the protein); thus, decreased rice intake likely explains the decrease in protein intake. Intakes of edible oils, a major contributor of fat in Bangladesh, have increased in Bangladesh (36), which could contribute to the trend of increasing fat intake. Despite increasing consumption of animal-sourced food (particularly fish, eggs, poultry, and dairy products) (39) and edible oil, the intake is insufficient to meet the recommended intake for the Bangladeshi people (37). This could explain the high insufficiency of protein and fat in Bangladesh.

In Bangladesh, agricultural diversity is associated with dietary diversity (40). Rice is overwhelmingly dominant in Bangladesh's crop patterns and diets. The growth in the production of non-rice crop and non-crop agricultural commodities (livestock and fish) must be augmented to improve the dietary diversity of the Bangladeshi population. Year-to-year price fluctuations are much larger for non-rice crops than for rice, indicating relatively high levels of market-induced risks for the production of non-rice crops. Developing value chains to link producers to food processing industries and food supermarkets can help mitigate these risks (7). Results of a randomized controlled trial called the Agriculture, Gender, and Nutrition Linkages project show that trainings that combined production of diverse, high-value, nutrient-rich foods and nutrition behavior change communication (BCC) were effective in improving production diversity and diet quality among rural farm households in Bangladesh (41). Broadly, it is essential to develop efficient and effective food systems for enhanced production of nutrient-rich food, as well as processing, marketing, preparation, and consumption of these foods. A study shows that Bangladeshi women are key actors within the food system, and that their empowerment improves dietary diversity as well as household food security (42).

While a sizeable proportion of rural Bangladeshi households produce agricultural commodities, most Bangladeshi rural households rely on markets for procuring various food items, including rice (which 53% of households report to have purchased in the previous week in 2018), *atta*(wheat flour) (94%), other cereals (81%), and various animal-sourced food items (between 71% and 80%) (**Supplemental Table 5**). Between 2011 and 2018, rural households’ own production slightly increased for rice, potatoes, and other vegetables, but decreased or was stagnate for many other food groups. Prices can inhibit access to healthy diets, particularly for poorer households. Evidence shows that the cost of a healthy diet is higher than the international poverty line (43), undermining access to healthy, balanced diets for economically disadvantaged households. In Bangladesh, 53% of the population spends less on food than the cost of the recommended diet. Specifically, individuals tend to overspend on staple and protein foods, and underspend on vegetables and dairy, likely due to the perishability of these particular items (8).

There are inequities in insufficient energy, protein, and fat intakes across the income distribution of households in our study population. Specifically, households from the lowest expenditure quintile show the highest insufficient intake for energy, protein, and fat, but also the highest excessive carbohydrate intake. One likely explanation is the inequity in food distribution and food consumption. Between 1985 and 2010, the reduction in the share of starches consumed was lower among the poorest (from 74% to 68%) than the richest groups (from 62% to 49%). Furthermore, the reduction in starches for the richest group was complemented with an increasing consumption of fish, meat, fruit, and beverages, which is relatively low for the poorest group (37). The proportion of meat, poultry, fish, and dairy product consumption among extremely poor households is nearly half that of non-poor households in the last 14 d (39).

The poor do not have adequate purchasing power to secure their access to nutritious foods, even when these foods are available in local markets. Creating effective demand for healthy foods requires employment and income generation among the poor. Promoting micro, small, and medium enterprises for food processing, packaging, storage, and transportation in the food value chains to deliver nutritious foods to consumers is a promising way to generate employment and income among the poor. Furthermore, promoting nutrition-sensitive social protection by integrating nutrition BCC has the potential to increase income as well as improve diets of the poor. Indeed, findings of a randomized controlled trial in rural Bangladesh reveal that cash transfers to ultra-poor women, when combined with high-quality nutrition BCC, increased household income and improved diets of children and adults, among other outcomes (44, 45).

This study has several strengths. The study used a large sample that is statistically representative of rural Bangladesh, focusing on the lifecycle approach of nutrient intake of all age groups except for children <2 y. Intakes collected from the previous 24 h excluded food wastage but included food consumed away from home. Energy and macronutrient intakes were estimated using the latest food-composition table of Bangladesh. A series of standardized steps was followed while calculating energy and macronutrients from food, including matching food descriptions between the food-composition table of Bangladesh and food consumption data and adjusting the edible coefficient of the food consumed in the household and the cooking methods of the food. However, this study also has limitations. Data on food intake were collected from the person responsible for cooking and distributing the food among household members, which could lead to possible measurement biases for individual intakes. Also, seasonality of food intake was not covered in this study.

In conclusion, dietary diversity has improved in Bangladesh in the last decade, but diets remain imbalanced and inequities in insufficient energy, protein, and fat intakes persist. Imbalanced diets and the uneven progress in energy and macronutrient intakes stress the need for coherent sets of policies and investments on a well-functioning food system and social protection to promote healthier and more equitable diets in Bangladesh.

## Supplementary Material

nxac198_Supplemental_File

## Data Availability

The data underlying this article are available in Harvard Dataverse. The BIHS 2012 data are available at https://doi.org/10.7910/DVN/OR6MHT. The BIHS 2018 data are available at https://doi.org/10.7910/DVN/NXKLZJ.

## Notes

Supported by the United States Agency for International Development (USAID) through Grant Number EEM-G-00-04-00013-00 and the CGIAR Research Program on Policies, Institutions, and Markets (PIM) led by the International Food Policy Research Institute (IFPRI). Freedom to publish the study findings was protected contractually in the agreement between the respective funding sources and IFPRI. All final decisions on the manuscript were made by the researchers.

Author disclosures: The authors report no conflicts of interest.

Supplemental Tables 1–5 are available from the “Supplementary data” link in the online posting of the article and from the same link in the online table of contents at https://academic.oup.com/jn/.

Abbreviations used: AMDR, Acceptable Macronutrient Distribution Range; AME, adult male equivalent; BCC, behavior change communication; BDHS, Bangladesh Demographic and Health Surveys; BIHS, Bangladesh Integrated Household Survey; BMR, basal metabolic rate; CIX, concentration Index; EER, estimated energy requirement; HIES, Household Income and Expenditure Surveys; PAL, physical activity level; PP, percentage point; PSU, primary sampling unit; SII, slope index of inequality.
