# Supplementary material for: Trends and Inequities in Food, Energy, Protein, Fat, and Carbohydrate Intakes in Rural Bangladesh
Source: J Nutr. 2022 Aug 30;152(11):2591–603. doi: 10.1093/jn/nxac198 (PMC9644183; doi:10.1093/jn/nxac198)
Supplement: nxac198_Supplemental_File [file nxac198_supplemental_file.docx]

**Supplemental Table 1. Equations for estimating BMR from body weight^1^**

|  | **BMR (kcal/d)** | |
| --- | --- | --- |
| **Age group, y** | **Male** | **Female** |
| <3 | 59.512 x weight – 30.4 | 58.317 x weight – 31.1 |
| 3-10 | 22.706 x weight + 504.3 | 20.315 x weight + 485.9 |
| 10-18 | 17.686 x weight + 658.2 | 13.384 x weight + 692.6 |
| 18-30 | 15.057 x weight + 692.2 | 14.818 x weight + 486.6 |
| 30-60 | 11.472 x weight + 873.1 | 8.126 x weight + 845.6 |
| ≥60 | 11.711 x weight + 587.7 | 9.082 x weight + 658.5 |

^1^Weight represents body weight in kg.

Source: (24)

**Supplemental Table 2. Quantity of food consumed in 2011 and 2018^1^**

| **Item** | **Desirable intake for Bangladeshi population^2^** | **2011** | **2018** | **Change from 2011 to 2018** | **p-value** |
| --- | --- | --- | --- | --- | --- |
|  | g/d | | | % |  |
| Rice | 350 | 448 | 379 | -15 | 0.0001 |
| Atta | 50 | 20.6 | 25.5 | 24 | 0.0015 |
| Other cereal |  | 9.9 | 8.7 | -12 | 0.1064 |
| Pulses | 50 | 9.0 | 14.1 | 57 | 0.0001 |
| Edible oils and fat | 30 | 17.7 | 24.9 | 41 | 0.0001 |
| Potatoes | 100 | 105 | 92.2 | -12 | 0.0001 |
| Green leafy vegetables | 100 | 35.0 | 31.0 | -11 | 0.0323 |
| Other vegetables | 200 | 177 | 167 | -6 | 0.0093 |
| Meat (beef, chicken, goat, etc.) | 40 | 14.2 | 19.6 | 38 | 0.0001 |
| Eggs | 30 | 4.9 | 10.1 | 106 | 0.0001 |
| Fish | 60 | 55.6 | 47.0 | -15 | 0.0001 |
| Milk and milk products | 130 | 17.4 | 28.0 | 61 | 0.0001 |
| Fruits | 100 | 8.3 | 20.2 | 143 | 0.0001 |
| Sugar/gur | 20 | 6.5 | 5.1 | -22 | 0.0004 |
| Total | 1240 | 929 | 873 | -6 | 0.0001 |

^1^Estimated from 24-hour recall individual dietary intake data.

^2^Desirable intakes for the Bangladeshi population were obtained from the *Desirable Dietary Pattern for Bangladesh* study, conducted by the Bangladesh Institute of Research and Rehabilitation in Diabetes, Endocrine and Metabolic Disorders (BIRDEM) (29). The total desirable dietary intake is 1,240 grams, which excludes spices.

**Supplemental Table 3. Percent contribution of rice and other food groups to protein, fat, and carbohydrate^1^**

^1^Estimated from 24-hour recall individual dietary intake data. Contribution from spices and other prepared food excluded.

^2^Asterisks indicate significant difference for changes between 2011 and 2018, *p<0.5, ** p<0.01, *** p<0.001

| **Food items** | **Protein** | | | **Fat** | | | **Carbohydrate** | | |
| --- | --- | --- | --- | --- | --- | --- | --- | --- | --- |
|  | **2011** | **2018** | **Change from 2011 to 2018** | **2011** | **2018** | **Change from 2011 to 2018** | **2011** | **2018** | **Change from 2011 to 2018** |
| Rice, *%* | 58.6 | 51.1 | -13.0***^2^ | 9.8 | 5.5 | -44.0*** | 85.0 | 80.2 | -6.0*** |
| Atta, *%* | 4.0 | 4.7 | 18.0** | 1.6 | 1.3 | -19.0** | 3.4 | 4.5 | 32.0*** |
| Other cereal, *%* | 1.2 | 1.1 | -8.0 | 0.1 | 0.1 | 0.0 | 1.6 | 1.7 | 6.0 |
| Pulses, *%* | 4 | 6.7 | 68.0*** | 0.6 | 0.8 | 33.0* | 1.1 | 1.8 | 64.0*** |
| Edible oils and fat, *%* | 0.0 | 0.0 | 0.0 | 64.1 | 68.9 | 7.0*** | 0.0 | 0.0 | 0.0 |
| Roots and tubers, *%* | 2.5 | 2.1 | -16.0*** | 0.8 | 0.4 | -50.0*** | 3.2 | 3.1 | -3.0 |
| Vegetables, *%* | 7.2 | 7.7 | 7.0*** | 2.0 | 1.5 | -25.0*** | 1.9 | 2.8 | 47.0*** |
| Meat (beef, chicken, goat, etc.), *%* | 3.7 | 5.6 | 51.0*** | 1.8 | 1.9 | 6.0 | 0.0 | 0.0 | 0.0 |
| Eggs, *%* | 1.2 | 2.5 | 108.0*** | 1.8 | 2.8 | 56.0*** | 0.0 | 0.0 | 0.0 |
| Fish, *%* | 14 | 12.3 | -12.0*** | 7.9 | 5.2 | -34.0*** | 0.0 | 0.0 | 0.0 |
| Milk and milk products, *%* | 0.9 | 1.6 | 78.0*** | 2.2 | 2.8 | 27.0*** | 0.2 | 0.3 | 50.0*** |
| Fruits, *%* | 0.2 | 0.3 | 50.0*** | 1.0 | 0.5 | -50.0*** | 0.2 | 0.5 | 150.0*** |
| Sugar/gur/sweet, *%* | 0.1 | 0.1 | 0.0 | 0.1 | 0.1 | 0.0 | 1.5 | 1.5 | 0.0 |

**Supplemental Table 4. Inequity gaps in insufficient intakes of energy, protein, and fat, and excessive intake of carbohydrate by sex, age group, and survey rounds**

|  |  |  | **Males** | | | | | | **Females** | | | | | |
| --- | --- | --- | --- | --- | --- | --- | --- | --- | --- | --- | --- | --- | --- | --- |
| **Indicators** | **Age group, y** | **Year** | **Q1**  **%** | **Q5**  **%** | **SII** | **SII p^2^** | **CIX** | **CIX p^2^** | **Q1**  **%** | **Q5**  **%** | **SII** | **SII p^2^** | **CIX** | **CIX p^2^** |
| **Energy, % insufficient** | 2-<5 | 2011 | 50.2 | 37.7 | -17.1*^1^ | 0.91 | -13.1** | 0.78 | 39.9 | 21.5 | -14.1 | 0.40 | -10.0* | 0.58 |
|  |  | 2018 | 46.7 | 30.7 | -16.1* |  | -11.3* |  | 47.9 | 37.0 | -23.3** |  | -13.8* |  |
|  | 5-<10 | 2011 | 21.0 | 13.6 | -11.7** | 0.03 | -8.7** | 0.04 | 14.4 | 15.2 | -3.6 | 0.09 | -4.2 | 0.15 |
|  |  | 2018 | 20.6 | 20.1 | 1.3 |  | -0.7 |  | 28.7 | 21.9 | -14.4** |  | -10.1** |  |
|  | 10-18 | 2011 | 23.4 | 12.1 | -14.9*** | 0.08 | -10.1*** | 0.09 | 30.9 | 15.4 | -19.6*** | 0.18 | -13.3*** | 0.36 |
|  |  | 2018 | 27.9 | 26.2 | -4.9 |  | -3.9 |  | 47.7 | 35.6 | -12.1** |  | -9.9*** |  |
|  | 19-40 | 2011 | 34.3 | 12.6 | -26.0*** | 0.03 | -17.5*** | 0.07 | 39.2 | 19.9 | -22.8*** | 0.001 | -15.5*** | 0.001 |
|  |  | 2018 | 37.9 | 24.6 | -15.6*** |  | -12.0*** |  | 34.3 | 27.6 | -8.2* |  | -6.3** |  |
|  | 41-60 | 2011 | 35.6 | 12.3 | -28.9*** | 0.05 | -19.3*** | 0.08 | 36.4 | 20.7 | -19.1*** | 0.03 | -12.2*** | 0.08 |
|  |  | 2018 | 43.0 | 27.2 | -18.1*** |  | -13.1*** |  | 31.2 | 24.3 | -6.6 |  | -5.6* |  |
|  | ≥61 | 2011 | 19.4 | 8.3 | -9.6 | 0.34 | -7.3* | 0.37 | 18.6 | 10.3 | -4.1 | 0.15 | -1.7 | 0.08 |
|  |  | 2018 | 25.7 | 19.4 | -1.9 |  | -2.7 |  | 28.0 | 13.0 | -14.9** |  | -10.3** |  |
| **Protein, % insufficient** | 2-<5 | 2011 | 26.3 | 7.4 | -17.4** | 0.84 | -11.8** | 0.95 | 28.3 | 9.0 | -26.5*** | 0.00 | -14.3*** | 0.003 |
|  |  | 2018 | 19.8 | 5.4 | -18.8** |  | -11.5** |  | 16.3 | 21.1 | 1.6 |  | 0.6 |  |
|  | 5-<10 | 2011 | 72.6 | 42.0 | -36.8*** | 0.76 | -22.9*** | 0.82 | 75.0 | 40.1 | -40.1*** | 0.34 | -24.5*** | 0.52 |
|  |  | 2018 | 67.1 | 38.1 | -39.3*** |  | -24.1*** |  | 71.1 | 29.5 | -46.5*** |  | -27.7*** |  |
|  | 10-18 | 2011 | 78.1 | 42.2 | -43.3*** | 0.64 | -27.5*** | 0.58 | 78.1 | 41.9 | -42.0*** | 0.82 | -27.0*** | 0.77 |
|  |  | 2018 | 73.5 | 31.8 | -46.2*** |  | -29.9*** |  | 71.1 | 33.2 | -40.8*** |  | -25.7*** |  |
|  | 19-40 | 2011 | 74.3 | 43.9 | -36.5*** | 0.64 | -23.4*** | 0.74 | 78.5 | 42.8 | 40.0*** | 0.27 | -24.9*** | 0.26 |
|  |  | 2018 | 69.9 | 35.0 | -39.1*** |  | -24.9*** |  | 72.3 | 33.5 | -44.7*** |  | -28.4*** |  |
|  | 41-60 | 2011 | 74.1 | 37.7 | -40.4*** | 0.51 | -25.5*** | 0.47 | 72.9 | 37.7 | -43.3*** | 0.64 | -27.1*** | 0.69 |
|  |  | 2018 | 73.1 | 34.3 | -44.2*** |  | -28.3*** |  | 75.2 | 40.2 | -41.3*** |  | -25.6*** |  |
|  | ≥61 | 2011 | 70.9 | 32.7 | -49.6*** | 0.31 | -31.0*** | 0.44 | 82.3 | 30.7 | -53.9*** | 0.05 | -35.4*** | 0.06 |
|  |  | 2018 | 70.0 | 36.5 | -41.5*** |  | -26.5*** |  | 66.0 | 33.8 | -36.4*** |  | -22.7*** |  |
| **Fat, % insufficient** | 2-<5 | 2011 | 99.2 | 72.8 | -23.7*** | 0.31 | -13.5*** | 0.51 | 98.8 | 90.9 | -9.9** | 0.12 | -5.6** | 0.08 |
|  |  | 2018 | 91.0 | 76.5 | -16.4*** |  | -10.7** |  | 95.1 | 67.3 | -19.8** |  | -12.4** |  |
|  | 5-<10 | 2011 | 99.7 | 91.6 | -10.9*** | 0.00 | -6.1*** | 0.00 | 99.7 | 95.6 | -5.3** | 0.00 | -3.0** | 0.00 |
|  |  | 2018 | 94.9 | 65.8 | -34.5*** |  | -22.2*** |  | 95.0 | 71.9 | -28.3*** |  | -17.3*** |  |
|  | 10-18 | 2011 | 99.8 | 92.5 | -8.3** | 0.005 | -4.1*** | 0.00 | 100.0 | 95.0 | -6.8** | 0.00 | -3.7** | 0.00 |
|  |  | 2018 | 97.5 | 82.8 | -19.4*** |  | -11.9*** |  | 98.7 | 81.3 | -21.7*** |  | -13.00*** |  |
|  | 19-40 | 2011 | 99.0 | 86.5 | -16.6*** | 0.01 | -9.6*** | 0.003 | 98.7 | 85.9 | -14.2*** | 0.00 | -8.6*** | 0.00 |
|  |  | 2018 | 91.6 | 67.6 | -26.0*** |  | -16.5*** |  | 94.1 | 69.0 | -31.4*** |  | -20.3*** |  |
|  | 41-60 | 2011 | 99.2 | 85.0 | -15.4*** | 0.00 | -9.1*** | 0.00 | 99.7 | 86.7 | -19.2*** | 0.002 | -11.2*** | 0.00 |
|  |  | 2018 | 93.8 | 66.9 | -35.6*** |  | -23.2*** |  | 95.9 | 71.4 | -32.2*** |  | -20.3*** |  |
|  | ≥61 | 2011 | 98.7 | 82.9 | -21.3*** | 0.60 | 11.7*** | 0.37 | 99.1 | 82.0 | -19.0*** | 0.76 | -10.7*** | 0.56 |
|  |  | 2018 | 91.8 | 71.7 | -25.0*** |  | 15.6*** |  | 91.1 | 67.4 | -21.1*** |  | -13.3*** |  |
| **Carbohydrate, % excessive** | 2-<5 | 2011 | 97.3 | 52.5 | -41.8*** | 0.59 | -25.0*** | 0.29 | 96.1 | 61.5 | -32.0*** | 0.36 | -18.6*** | 0.20 |
|  |  | 2018 | 85.4 | 47.0 | -46.6*** |  | -31.3*** |  | 86.3 | 43.3 | -39.5*** |  | -25.5*** |  |
|  | 5-<10 | 2011 | 99.7 | 81.9 | -22.1*** | 0.00 | 12.0*** | 0.00 | 99.3 | 86.7 | -15.2*** | 0.00 | -8.8*** | 0.00 |
|  |  | 2018 | 90.8 | 50.1 | -45.9*** |  | -29.8*** |  | 93.5 | 51.6 | -43.8*** |  | -27.6*** |  |
|  | 10-18 | 2011 | 99.7 | 84.1 | -19.3*** | 0.009 | -9.7*** | 0.00 | 100.0 | 88.3 | -16.4*** | 0.001 | -8.5*** | 0.00 |
|  |  | 2018 | 94.2 | 68.0 | -33.9*** |  | -21.2*** |  | 96.5 | 71.6 | -30.5*** |  | -19.7*** |  |
|  | 19-40 | 2011 | 99.2 | 89.5 | -14.0*** | 0.001 | -8.1*** | 0.00 | 99.3 | 87.5 | -13.8*** | 0.00 | -7.7*** | 0.00 |
|  |  | 2018 | 95.7 | 71.1 | -25.9*** |  | -15.7*** |  | 97.2 | 75.0 | -28.2*** |  | 17.4*** |  |
|  | 41-60 | 2011 | 100.0 | 86.8 | -17.2*** | 0.01 | 8.7*** | 0.00 | 100.0 | 89.9 | -16.2*** | 0.004 | -8.7*** | 0.00 |
|  |  | 2018 | 96.4 | 74.2 | -29.3*** |  | -18.2*** |  | 97.6 | 76.7 | -27.6*** |  | -17.0*** |  |
|  | ≥61 | 2011 | 100.0 | 82.6 | -23.0*** | 0.51 | 11.9*** | 0.23 | 100.0 | 83.9 | -20.0*** | 0.75 | -10.8*** | 0.50 |
|  |  | 2018 | 97.4 | 75.2 | -27.8*** |  | -16.6*** |  | 94.7 | 70.7 | -22.2*** |  | -13.6*** |  |

^1^Asterisks indicate significant difference for inequity between Q1 and Q5, *** p<0.001, ** p<0.01, *p<0.5.

^2^p-values for difference between 2011 and 2018.

CIX, Concentration Index; Q, quintile; SII, Slope Index of Inequality.

**Supplemental Table 5. Source of food items consumed in the last 7 days by survey round^1^**

| **Food Item** | **Purchased** | | | **Own Production** | | | **Other Sources** | | |
| --- | --- | --- | --- | --- | --- | --- | --- | --- | --- |
|  | **2011** | **2018** | **Change from 2011 to 2018** | **2011** | **2018** | **Change from 2011 to 2018** | **2011** | **2018** | **Change from 2011 to 2018** |
| Rice, *%* | 59.8 | 52.5 | -7.3***^2^ | 38.0 | 43.2 | 5.2*** | 2.2 | 4.4 | 2.2*** |
| Atta, *%* | 89.0 | 94.1 | 5.1** | 4.8 | 2.8 | -2.0 | 6.2 | 3.1 | -3.1*** |
| Other cereal, *%* | 68.0 | 81.4 | 13.4*** | 25.0 | 13.1 | -11.9*** | 7.0 | 5.4 | -1.6** |
| Pulses, *%* | 89.8 | 90.5 | 0.7 | 6.1 | 5.4 | -0.7 | 4.1 | 4.1 | 0.0 |
| Edible oils and fat, *%* | 98.5 | 98.4 | -0.1 | 1.1 | 1.0 | -0.1 | 0.5 | 0.6 | 0.1 |
| Potatoes, *%* | 94.8 | 87.7 | -7.1*** | 3.8 | 7.8 | 4.0*** | 1.4 | 4.5 | 3.1*** |
| Green leafy vegetables, *%* | 46.7 | 43.2 | -3.5* | 21.9 | 21.0 | -0.9 | 31.4 | 35.8 | 4.4** |
| Other vegetables, *%* | 92.1 | 87.8 | -4.3*** | 5.1 | 7.6 | 2.5*** | 2.9 | 4.6 | 1.7*** |
| Meat (beef, chicken, goat, etc.), *%* | 66.1 | 79.1 | 13.0*** | 18.9 | 13.6 | -5.3*** | 15.1 | 7.3 | -7.8*** |
| Eggs, *%* | 59.6 | 68.8 | 9.2*** | 38.7 | 29.9 | -8.8*** | 1.7 | 1.3 | -0.4 |
| Fish, *%* | 79.7 | 80.0 | 0.3 | 7.3 | 6.6 | -0.7 | 12.9 | 13.4 | 0.5 |
| Milk and milk products, *%* | 64.6 | 71.2 | 6.6*** | 29.4 | 21.4 | -8.0*** | 6.1 | 7.4 | 1.3 |
| Fruits, *%* | 49.1 | 50.9 | 1.8 | 20.6 | 17.8 | -2.8** | 30.3 | 31.4 | 1.1 |
| Sugar/gur, *%* | 97.2 | 97.8 | 0.6 | 1.2 | 0.5 | -0.7** | 1.6 | 1.7 | 0.1 |
| Salt, *%* | 100.0 | 100.0 | 0.0 | 0.0 | 0.0 | 0.0 | 0.0 | 0.0 | 0.0 |
| Spices and Condiments, *%* | 95.9 | 97.0 | 1.1* | 2.8 | 1.9 | -0.9** | 1.3 | 1.2 | -0.1 |

^1^ Estimated from the last 7 days recall

^2^Asterisks indicate significant difference for changes between 2018/19 and 2011/12, *p<0.5, ** p<0.01, *** p<0.001
